# Endostatin, an Inhibitor of Angiogenesis, Decreases After Bidirectional Superior Cavopulmonary Anastamosis

**DOI:** 10.1007/s00246-012-0441-2

**Published:** 2012-09-08

**Authors:** Aida Field-Ridley, Ritva Heljasvaara, Taina Pihlajaniemi, Ian Adatia, Christine Sun, Roberta L. Keller, Wen Hui Gong, Sanjeev Datar, Peter Oishi, Jeffrey R. Fineman

**Affiliations:** 1 Department of Pediatrics, University of California, San Francisco, CA USA; 2 Oulu Center for Cell Matrix Research, Oulu, Finland; 3 Department of Pediatrics, University of Alberta, Alberta, Canada; 4 Cardiovascular Research Institute, University of California, San Francisco, CA 94143-0106 USA; 5 Department of Pediatrics, University of California, Davis, 2516 Stockton Avenue, Sacramento, CA 95817 USA

**Keywords:** Angiogenesis inhibitor, Cardiac repair, Collagen XVIII, Congenital heart disease, Endostatin, Pulmonary arteriovenous malformations

## Abstract

Pulmonary arteriovenous malformations (PAVMs) are a common source of morbidity after bidirectional superior cavopulmonary anastomosis (Glenn). The diversion of hepatic venous effluent away from the pulmonary circulation after Glenn appears to play a significant role in the pathogenesis of PAVMs. Although the liver is known to produce factors that regulate vascular development, specific hepatic inhibitors of angiogenesis have not been described in the post-Glenn population. Endostatin, produced from its precursor collagen XVIII, is a potent inhibitor of angiogenesis produced by the liver. This study aimed to investigate the hypothesis that endostatin levels decrease in patients after Glenn. Levels of endostatin and its precursor, long-type collagen XVIII, were determined by enzyme-linked immunoassay and immunoprecipitation, respectively, for serum samples from 38 patients undergoing Glenn, total cavopulmonary anastomosis (Fontan), or biventricular repair of cardiac defects. Samples were obtained before surgery and 24 h afterward. In patients undergoing a bidirectional Glenn procedure, endostatin levels decreased after surgery (*n* = 17; 4.42 vs 3.34 ng/ml; *p* < 0.001), and long type-collagen XVIII levels increased by 200 % (*n* = 10; *p* = 0.0001). However, endostatin levels did not change after surgery in patients undergoing Fontan (*n* = 13) or biventricular repair (*n* = 8). In patients undergoing Fontan, long-type collagen XVIII increased by 18 % (*p* < 0.01), whereas in control subjects, the levels were unchanged. These data suggest that the diversion of hepatic blood flow away from the pulmonary circulation in patients after the Glenn procedure inhibits endostatin production from collagen XVIII, resulting in decreased circulating serum endostatin levels. A decrease in endostatin may promote angiogenesis. The mechanism whereby the pulmonary circulation processes endostatin and its potential role in the pathogenesis of PAVMs warrant further study.

Pulmonary arteriovenous malformations (PAVMs) that develop after superior cavopulmonary anastomosis or bidirectional Glenn surgery are an important cause of morbidity secondary to progressive cyanosis in the population with complex congenital heart disease [1]. The proportion of patients who experience PAVM ranges from 35 to 100 %, depending on the mode and timing of detection [24].

The mechanism by which PAVMs arise is not understood. It has been recognized for the past 15 years that diversion of hepatic venous effluent away from the lung or impaired hepatic function plays a significant role in the pathogenesis of PAVMs [19]. Furthermore, structural similarities exist between PAVMs developing after bidirectional Glenn and the hepatopulmonary syndrome (HPS) from biliary and liver disease [7]. Specifically, there are dilated precapillary and capillary pulmonary blood vessels, increased microvessel density, and animal model data consistent with abnormal vascular remodeling [20, 23]. Alterations in pro-angiogenic factors [e.g., upregulation of vascular endothelial growth factor (VEGF) receptors and increased circulating VEGF levels] have been described in association with PAVMs in children who have undergone bidirectional Glenn [21, 22]. Although VEGF is a known circulating pro-angiogenic factor [5], the role of hepatic-specific regulators of angiogenesis and their exclusion from the pulmonary circulation in the development of PAVMs after bidirectional Glenn are unknown.

The liver participates in regulating angiogenesis [2]. Endostatin, a potent inhibitor of angiogenesis, is the 20-kDa protein from the C-terminus of collagen XVIII [14]. The long isoform of collagen XVIII is hepatocyte specific [13]. This study aimed to describe and compare changes in endostatin and its precursor collagen XVIII in patients who had undergone Glenn and total cavopulmonary anastomosis (Fontan) with those of patients who had complete biventricular repair of congenital heart disease.

## Materials and Methods

From a prior institutional review board (IRB)-approved study, 38 plasma samples were obtained from patients undergoing bidirectional Glenn, modified Fontan operation, or biventricular repair (Table 1). Samples were obtained from a systemic artery before the respective surgical procedure and 24 h afterward.

**Table 1:** Clinical characteristics and demographics of the study patients

	Glenn (*n* = 17)	Fontan (*n* = 13)	Control (*n* = 8)
Gender: M:F (%)	10:7 (59)	8:5 (62)	6:2 (75)
Age (median, IQR)	5 months (4, 11)	5 years (4, 6)	0.3 months (0.3, 3)
Lesion (*n*)	DORV (3)	DORV (5)	AVSD (3)
	d-TGA (3)	HLHS (2)	d-TGA (3)
	Heterotaxy (3)	DILV (2)	TOF (2)
	HLHS (2)	Other (4)	
	Other (6)		

*IQR* interquartile range, *DORV* double-outlet right ventricle, *AVSD* atrioventricular septal defect, *d-TGA* d-transposition of the great arteries, *HLHS* hypoplastic left heart syndrome, *DILV* double-inlet left ventricle, *TOF* tetralogy of Fallot

### Endostatin Analysis

We determined endostatin levels by enzyme-linked immunoassay (ELISA) (R & D Systems, Inc., Minneapolis, MN). The assessed interassay precision of the kit used demonstrated a coefficient of variation between 3.6 and 6.9 %. Intraassay precision demonstrated a coefficient of variation between 5.7 and 7.9 %. Plasma samples analyzed in this study were thawed from −80 °C to room temperature. Measurements were performed in duplicate at 1:50 and 1:100 dilutions. Measurement variability was minimal and comparable with that reported in the kit.

### Collagen XVIII Analysis

Long-type collagen XVIII was determined by previously published methods [17]. Briefly, plasma samples were immunoprecipitated with the monoclonal anti-all type XVIII antibody, DB144-N2, bound to affinity-purified antimouse immunoglobin G (IgG)-coated magnetic beads (Dynabeads M-280; Dynal, Oslo, Norway). The DB144-N2 antibody was incubated with antimouse IgG-coated beads in phosphate-buffered saline (PBS) buffer (pH 7.4) containing 0.1 % bovine serum albumin (BSA) for 2 h at room temperature.

After several washes in PBS, 100 μl of human plasma was incubated with the DB144-N2 antibody–coated beads overnight at 4 °C. Unbound proteins were removed by washing with PBS containing 1 % Triton-X100 and 0.1 % sodium dodecyl sulfate (SDS). Specifically, bound polypeptides were eluted by boiling for 3 min in SDS sample buffer containing 5 % 2-mercaptoethanol, resolved by 7–12 % SDS-polyacrylamide gel electrophoresis (PAGE), and detected by Western blot with anti-LONG variant antibody (QH 1415).

### Western Blot Analysis

A 20-μl protein sample was loaded onto SDS-PAGE, electrotransferred to a polyvinylidene fluoride (PVDF) membrane (Immobilon; Millipore Billerica, MA), and probed with rabbit polyclonal antibodies against long-type collagen XVIII at a concentration of 1 μg/ml in 1 % fat-free milk powder followed by goat antirabbit antibody. After extensive washing of the membrane with 1 × PBS 0.1 % Tween, proteins reactive to QH 1415 were visualized with enhanced chemiluminescence (ECL) Western blotting detection reagents (Amersham Pharmacia Biotech, Amersham, UK).

### Statistical Analysis

Frequencies were tabulated for categorical variables. Changes in continuous variables not normally distributed within groups were compared using the paired Wilcoxon signed-rank test. Differences in continuous variables between two groups not normally distributed were compared with the Wilcoxon rank-sum test.

## Results

All the groups had a preponderance of males (Table 1). The age ranges were typical for the stage of palliation, whereas the control group undergoing biventricular repair was significantly younger. The double-outlet right ventricle (DORV) was the predominant lesion in the palliated groups, whereas the lesions in the control group were atrioventricular septal defect (AVSD), d-transposition of the great arteries (d-TGA), and tetralogy of Fallot (TOF).

**Fig. 1:**
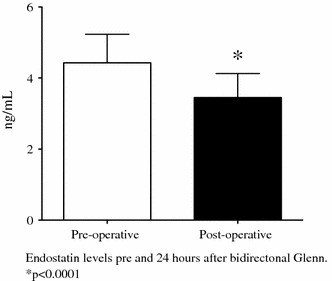
Pre- and postoperative plasma endostatin levels in 17 infants undergoing superior cavopulmonary anastomosis (Glenn). Endostatin levels decreased after the Glenn. Values are mean ± standard deviation. *p < 0.0001 vs preoperative levels

In the patients undergoing Glenn, endostatin levels decreased postoperatively compared with preoperative levels (4.42 ng/ml; 95 % confidence interval [CI], 4.12–4.70 vs 3.34 ng/ml; 95 % CI, 3.21–3.69 ng/ml; *p* < 0.0001) (Fig. 1). Conversely, in the patients undergoing modified Fontan, pre- and postoperative endostatin levels did not differ (*p* = 0.3).

**Fig. 2:**
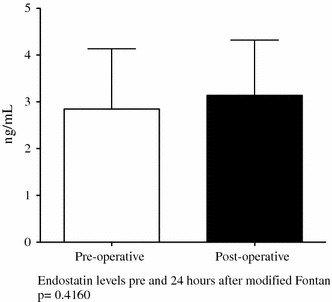
Pre- and postoperative plasma endostatin levels in 13 patients undergoing total cavopulmonary anastomosis (Fontan). Endostatin levels did not change postoperatively after the Fontan. Values are mean ± standard deviation

**Fig. 3:**
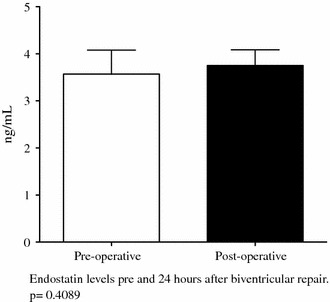
Pre- and postoperative plasma endostatin levels in 8 patients undergoing biventricular repair. Endostatin levels did not change postoperatively after biventricular repair. Values are mean ± standard deviation

The patients in the control group undergoing biventricular repair showed a trend toward increasing endostatin levels after surgery (*p* = 0.07) (Figs. 2, 3). The preoperative endostatin levels in the Glenn group were higher than in the modified Fontan and control groups. In addition, the endostatin levels after bidirectional Glenn continued to decrease at the time of the Fontan (3.45 ± 0.7 ng/ml; 95 % CI, 3.21–3.69 vs 2.8 ± 1.2 ng/ml; 95 % CI, 2.3–3.4 ng/ml; *p* = 0.03).

**Fig. 4:**
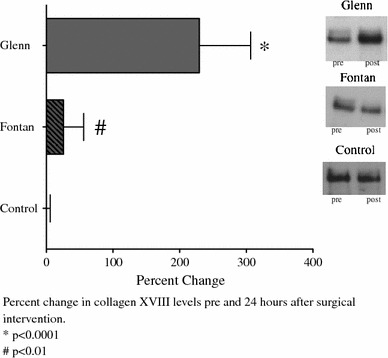
Pre- and postoperative plasma long-type collagen XVIII levels (via immunprecipitation) in patients undergoing superior cavopulmonary anastomosis (Glenn, *top*, *n* = 10), total cavopulmonary anastomosis (Fontan, *middle*, *n* = 7), and biventricular repair (control, *bottom*, *n* = 4). Representative blots are shown. Long-type collagen XVIII levels increased more than twofold after Glenn and 18 % after Fontan but did not change after biventricular repair. Values are percentage of change from preoperative values ± standard deviation. *p < 0.05 vs preoperative levels

In the patients undergoing bidirectional Glenn, the long-type collagen XVIII levels, as determined by immunoprecipitation, increased postoperatively compared with preoperative levels (optical density: 74,800 [95 % CI, 438,955–1,934,975] vs 2,148,312 [95 % CI, 862,227–5,158,851] + 200 % [*p* < 0.0001]) (Fig. 4).

In the patients undergoing modified Fontan, the long-type collagen XVIII levels also increased postoperatively, although to a lesser extent than in the group undergoing bidirectional Glenn (optical density: 434,232 [95 % CI, 204,743.6–663,721.5] vs 440,236 (95 % CI, 211,719–668,754] + 18 % [*p* < 0.01]) (Fig. 4). In the control group, the pre- and postoperative collagen XVIII levels did not differ.

## Discussion

Since the discovery of endostatin in 1997, its anti-angiogenic properties have been used to treat many types of solid tumors in cancer patients with varying success [10]. Endostatin, the 20-kDa C-terminus of collagen XVIII, is known to inhibit the VEGF pathway via the competitive inhibition of the VEGFR2 receptor, induce apoptosis via the bcl-2 pathway, and inhibit endothelial cell migrations via blockade of basic fibroblast growth factor (bFGF) [3, 8, 9, 14, 16]. Its precursor, collagen XVIII, exists both as a component of the basement membrane and as a circulating plasma protein. It exists in short, intermediate, and long isoforms. The short isoform is ubiquitously present in the basement membrane of various tissues, whereas the long type seems specific to hepatocytes and liver sinusoids [18].

The biologic roles of collagen XVIII are largely unknown, but deficiencies have been linked to autosomal-recessive Knobloch disease, which includes abnormalities in the vascularization of the retina and encephaloceole formation [12]. To date, the pathophysiology of long-type collagen XVIII deficiency has not been described in vivo.

Our data demonstrate significant perioperative changes in both endostatin and the hepatocyte-specific long-type collagen XVIII. Endostatin decreases, whereas its precursor collagen XVIII increases within 24 h after bidirectional Glenn. These findings suggest that the decrease in endostatin is not caused by the decreased hepatic production of its precursor but rather by a potential perturbation in its cleavage, such as first-pass metabolism by the lungs. The mechanism for these novel changes is unknown but warrants further investigation.

First-pass metabolism by the lung has been described in other enzymatic processes, including clearance of natriuretic peptides and angiotensin-converting enzyme (ACE). The influence of hepatic venous drainage on pulmonary metabolism in humans is unclear. However, in an ovine experimental model of the classic Glenn procedure (i.e., anastomosis of the superior vena cava to one divided pulmonary artery), PAVMs developed reliably on the ipsilateral side within 8 weeks. The development of these PAVMs was associated with a decrease in ACE activity and expression over the first 4 weeks before a subsequent return to baseline at the end of 8 weeks [11]. These experimental data provide a framework in which to investigate how the liver may influence pulmonary vascular metabolism.

Endostatin is released in all isoforms of collagen XVIII by at least two known pathways. Matrix metalloproteinase and elastase can release endostatin in a two-step process [4, 6, 26]. In addition, cleavage of endostatin can occur in a single step by cathepsin L [4, 25]. Regulation of these enzymes and the conditions in which they are active or become inactivated are largely unknown. The data in the literature are conflicting as to whether hypoxia increases or decreases endostatin in different animal models [15, 25]. The data suggest, however, that oxygen tension may influence the enzymatic release of endostatin. Interestingly, both the basement membrane form, the short isoform, and the circulating or long isoform release endostatin via the same enzymatic pathway. This suggests that endostatin may play a role in both local and systemic inhibition of angiogenesis [26].

The circulating endostatin levels in patients undergoing the Glenn procedure for palliation of single-ventricle heart disease decrease postoperatively. This is associated with an increase in its precursor, collagen XVII.

Whether these changes represent the fact that endostatin may normally undergo first-pass metabolism in the lungs, which then becomes disrupted after Glenn, is unknown but warrants investigation. We suggest that the decrease in endostatin, an inhibitor of angiogenesis, may contribute to the development of clinically important PAVMs. However, important limitations of this study include its retrospective design, precluding our ability to correlate the incidence of PAVM with the change in endostatin levels. In addition, the amount of plasma available was limited, which prevented the analyses of collagen XVIII in all patients and other hepatic regulators of angiogenesis. Therefore, confirmatory studies of these initial provocative findings are needed.

In addition, a prospective longitudinal study to assess the role of endostatin derived from hepatic-specific collagen XVIII in the pathogenesis of PAVM is warranted and may result in important prevention or treatment strategies to limit cyanosis and promote the longevity of the bidirectional Glenn as a palliative procedure. This would have importance not only in the management of patients with congenital heart disease unsuitable for biventricular repair or further palliation with a modified Fontan operation but also for patients with hepatopulmonary syndrome awaiting a liver transplant.
